# Watching what others eat: exposure to social-media eating content, body appreciation, and eating behaviors in Turkish adults

**DOI:** 10.3389/fpubh.2026.1963635

**Published:** 2026-10-22

**Authors:** Emre Batuhan Kenger, Tuğçe Özlü Karahan, Aslı Başak Toprak, İpek Arslan, Tuğba Sarıtaş, Zeynep Özgen, Hatice Merve Bayram, Arda Ozturkcan

**Affiliations:** 1 Department of Nutrition and Dietetics, Faculty of Health Sciences, Fenerbahçe University, Istanbul, Türkiye; 2 Department of Nutrition and Dietetics, Hamidiye Faculty of Health Sciences, University of Health Sciences Türkiye, Istanbul, Türkiye; 3 Department of Nutrition and Dietetics, Faculty of Health Sciences, Istanbul Bilgi University, Istanbul, Türkiye; 4 Department of Nutrition and Dietetics, Faculty of Health Sciences, Istanbul Gelisim University, Istanbul, Türkiye

**Keywords:** body appreciation, cognitive restraint, eating behavior, emotional eating, food-related videos, social media, uncontrolled eating

## Abstract

**Background:**

It remains unclear whether eating behaviors are associated with overall social media use or specifically with the duration and genre of food-related video exposure. This study examined the associations of general social media use, food-video viewing duration, most-watched food-video genre, and body appreciation with eating behaviors among adults living in Istanbul, Türkiye.

**Methods:**

This cross-sectional study included 364 (75.5% female, the mean age of 29.3 ± 10.8 years) adults residing in Istanbul, Türkiye between March-May 2026. Data were collected through an online questionnaire comprising social media and food-video viewing patterns, eating behaviors using the Three-Factor Eating Questionnaire-R21, and body appreciation using the Body Appreciation Scale-2. Data was analyzed using SPSS 24.0.

**Results:**

Overall social media use was not independently associated with uncontrolled eating (β = 0.035, *p* = 0.517), emotional eating (β = −0.030, *p* = 0.566), or cognitive restraint (β = −0.055, *p* = 0.290). In the primary adjusted models, compared with viewing food-related videos for 0–1 h/day, viewing for 2–4 h/day was associated with higher uncontrolled eating (β = 0.208, *p* < 0.001), emotional eating (β = 0.161, *p* = 0.001), and cognitive restraint (β = 0.103, *p* = 0.045). However, the cognitive-restraint association for this category was not statistically significant after expanded covariate adjustment or in the non-linear sensitivity analysis, indicating a model-dependent finding. Viewing for >4 h/day was associated with higher emotional eating (β = 0.118, *p* = 0.001) and cognitive restraint (β = 0.147, *p* = 0.006), but not uncontrolled eating. Higher body appreciation was independently associated with lower uncontrolled eating (β = −0.212, *p* < 0.001) and emotional eating (β = −0.191, *p* < 0.001). The final models had adjusted R^2^ values of 0.137, 0.197, and 0.122, respectively.

**Conclusions:**

Food-video viewing duration was associated with uncontrolled and emotional eating, while associations with cognitive restraint varied across model specifications. Greater body appreciation was independently associated with lower uncontrolled and emotional eating.

## Introduction

Eating has increasingly become something people watch. Across Instagram, TikTok, and YouTube, hosts consume multiple portions on camera, 30-second reels compress an entire recipe from batter to finished cake, reviewers guide viewers around restaurant tables, and creators document everything they eat in a day (1, 2). Turkish audiences are particularly well positioned to encounter such content. In 2025, 90.9% of individuals aged 16–74 used the internet, while YouTube and Instagram—the two platforms on which food-related videos circulate most widely—reached 72.9% and 68.1% of internet users, respectively (3). Over the past decade, this transformation appears to reflect displacement rather than simple addition. Between 2015 and 2025, the proportion of individuals aged 10 years and older who reported spending time on social media more than doubled, rising from 33.9% to 71.7%, whereas the proportion reading newspapers or magazines declined from 39.4% to 20.1% (4). Recommendation systems may further amplify this shift by prioritizing engagement: once viewers pause on or interact with food-related content, they are likely to be shown more of it (5).

Watching food-related content is not neutral for appetite. Boswell and Kober (6) found that food images and videos elicited cravings and predicted subsequent intake, with effects comparable to exposure to real food and greater than those observed for odor cues. Cue-elicited craving also prospectively predicted weight gain. Repeatedly pairing visual cues with palatable foods may transfer incentive value to those cues, allowing them to trigger food seeking even in the absence of physiological hunger (7, 8). This appetitive drive is shaped by the food environment people inhabit: among emerging adults, hedonic hunger and perceived characteristics of the obesogenic environment were jointly associated with higher ultra-processed food consumption (9). Social media arguably extends that cue environment into a setting users carry with them and revisit throughout the day.

However, food-related content may influence eating through pathways beyond appetite stimulation. Viewers may use videos of others eating as a source of companionship, distraction, or relief from eating alone (10, 11), suggesting an affect-regulation pathway consistent with emotional eating (12). Conversely, fitness- and diet-oriented content may promote restrictive eating by communicating dietary rules, idealized portion norms, and moral judgements about food. Restraint shaped by such prescriptive messaging may resemble rigid rather than flexible dietary control (13). Thus, food-related social media content may simultaneously intensify cue-driven eating, serve emotional functions, and reinforce restrictive dietary norms.

Evidence for these routes is uneven across genres. Mukbang has received the most attention, and the findings are mixed. Among Turkish university students, mukbang addiction scores were higher among those who screened positive for disordered eating and were positively associated with uncontrolled and emotional eating, although the hypothesized mediation effect was not supported (14). Among Korean adults, meanwhile, more frequent mukbang viewing was associated with both better dietary balance and poorer dietary moderation within the same sample (15). Research on food-diary and fitness content has focused primarily on body-image-related outcomes; for example, viewing “what I eat in a day” videos has been shown to influence dieting intentions and body evaluations among young adults (16). By contrast, restaurant reviews and recipe videos—which together accounted for half of the most frequently watched content in the present sample—have received almost no attention in behavioral research. The applicability of the existing evidence to adults is further limited by two recurring design features: samples consist predominantly of adolescents or university students, and exposure is typically measured as total time spent on social media rather than engagement with food-related content specifically. Sanzari et al. (17) demonstrated the importance of this distinction: the type of content consumed predicted body-image disturbance and binge eating, whereas time spent online and the number of platforms used did not.

Most research in this area has focused on risk factors, whereas body appreciation represents a potentially protective pathway. It refers to accepting and holding a favorable view of one's body while rejecting media-promoted appearance ideals as the sole standard of beauty. A defining feature is protective filtering—the selective processing of body-related information and rejection of messages perceived as harmful (18). Within the acceptance model of intuitive eating, body appreciation may help individuals respond to environmental influences while continuing to eat according to internal hunger and satiety cues rather than external prompts (19). Its protective relevance is also supported longitudinally: in an 8-month prospective study, body appreciation predicted the continued absence of core eating-disorder symptoms among women who were initially asymptomatic (20). Yet, within the social media literature, body appreciation has been examined almost exclusively as an outcome adversely affected by exposure. Whether it also contributes independently to explaining eating behavior when modeled alongside social media exposure remains unclear.

Taken together, these observations can be situated within a multidomain perspective in which digital exposure, psychological characteristics, and nutrition-related behaviors are interconnected but conceptually distinct. Food-related social media content represents a digital environmental exposure that may combine appetitive food cues with appearance ideals, dietary norms, and socially transmitted messages about eating (2, 5). Psychological characteristics such as body appreciation may be relevant to how individuals interpret and respond to such content, while uncontrolled eating, emotional eating, and cognitive restraint represent distinct eating-behavior correlates within the nutrition-related behavioral domain. Recent reviews similarly indicate that eating-related social media content is associated with both body-image outcomes and eating behaviors, although the direction and magnitude of these associations vary according to content type and study design (21, 22). Therefore, this study aimed to examine the associations of general social media use, time spent watching food-related videos, most frequently watched video genre, and body appreciation with eating behaviors.

## Methods

### Study design and participants

This descriptive cross-sectional study was conducted on adults living in Istanbul, Türkiye between March-May 2026. Participants were recruited using non-probability convenience and snowball sampling. The questionnaire was administered through Google Forms, and the survey link was shared on Facebook, Twitter, and Instagram. Email addresses were used to check for duplicate responses. Data were collected using an online, self-administered questionnaire that included items on sociodemographic, lifestyle, anthropometric, and health-related characteristics; patterns of general social media use and food-related video consumption; perceived responses to food-related social media content; the Three-Factor Eating Questionnaire-R21 (TFEQ-R21); and the Body Appreciation Scale-2 (BAS-2). Self-reported body weight and height were used to calculate body mass index.

The sample size was estimated a priori using GPower version 3.1.9.7. Based on previous research reporting a positive correlation between problematic mukbang watching and disordered eating (10), a two-tailed bivariate correlation analysis with an effect size of r = 0.24, α = 0.05, and 95% statistical power indicated a minimum required sample size of 219 participants. In addition, because the primary analyses involved hierarchical multiple regression, a sensitivity analysis was performed using GPower. With the achieved sample size of 364 participants, 10 predictors, α = 0.05, and 95% power, the analysis indicated that the study was sufficiently powered to detect an overall regression effect as f^2^ = 0.069.

Participants were eligible to participate if they were aged ≥18 years, lived in Istanbul, Türkiye, were able to read and understand Turkish, actively used at least one social media platform, watched food- or nutrition-related social media videos, and provided electronic informed consent. Eligibility and exclusion criteria, including the restrictive-diet criterion, were established before the study began and before examination of the outcome data. These criteria were assessed using questionnaire responses during data cleaning after data collection. All exclusion decisions were completed before the TFEQ-R21 and BAS-2 scores were calculated. Respondents who submitted the survey despite meeting an exclusion criterion were removed from the analytical sample. Exclusion criteria comprised a self-reported diagnosed eating disorder, current restrictive dieting, unavailable or clearly implausible anthropometric data, and incomplete responses to required scale items. Eating-disorder diagnosis was based on self-report, without independent clinical verification. Unavailable anthropometric data comprised responses of “I do not know” for body weight and/or height. Clearly implausible anthropometric entries included values such as a height of 2,000 m or a body weight of 1,500 kg; no corrected values were inferred. High body weight alone was not an exclusion criterion. Incomplete scale responses comprised unanswered required items in the TFEQ-R21 and/or BAS-2, rather than contradictory questionnaire responses. The three participants excluded for restrictive dieting reported a crash diet consisting only of vegetable dishes at lunch and dinner, with no other foods consumed. This dietary pattern was identified during data cleaning as meeting the pre-established restrictive-diet exclusion criterion.

A total of 385 individuals submitted the survey. Of these, 21 were excluded from the analyses: six because they answered “I do not know” for body weight and/or height, four because they reported clearly implausible anthropometric values, six because of unanswered required scale items, three because they met the restrictive-diet exclusion criterion, and two because of a self-reported diagnosed eating disorder. The final analytical sample comprised 364 participants, all of whom reported watching food- or nutrition-related social media videos.

### Sociodemographic characteristics, anthropometric measurements, social media use, and food-related content

Sociodemographic, lifestyle, and health-related information, including age, sex, education, marital and employment status, personal income, living arrangement, chronic disease status, smoking, alcohol use, and physical activity level, was collected using self-reported questionnaire items. Physical activity was assessed using a single descriptive item with five response categories: sedentary (sitting throughout the day and no regular exercise), light (short walks or light movement during the day), moderate (regular walking, housework, or physical activity several times a week), active (moderate-intensity exercise on 3–5 days per week), and very active (being continuously active in daily life or regularly undertaking vigorous exercise). Smoking and alcohol use were each assessed using a yes/no question, with a “yes” response indicating current use at the time of the survey. Body weight (kg) and height (cm) were self-reported, and body mass index (BMI) was calculated as body weight in kilograms divided by height in meters squared (kg/m^2^). Participants were also asked about their most frequently used social media platform, daily social media use, primary purpose of use, daily time spent watching food-related videos, and the food-video genre they watched most frequently. Perceived responses to food-related social media content were assessed using a series of researcher-developed statements rated on a five-point Likert scale: strongly disagree, disagree, neither agree nor disagree, agree, and strongly agree. The researcher-developed items were reviewed by the author team, whose members have expertise in nutrition and dietetics, for relevance to the study objectives, clarity, and comprehensibility. No separate pilot testing or formal quantitative assessment of content validity was conducted. Therefore, these items were analyzed individually as exploratory items rather than combined into a validated psychometric scale. Daily social media use and food-video viewing duration were self-reported for a typical day by selecting one of the response categories defined a priori in the questionnaire. Daily social media use categories were < 1, 1–2, 2–4, 4–6, and >6 h/day, whereas food-video viewing categories were 0–1, 2–4, and >4 h/day. Weekdays and weekends were not assessed separately.

The food-video viewing options were presented in the questionnaire as 0–1, 2–4, and >4 h/day. These labels leave an apparent gap between 1 and 2 h/day, and the questionnaire did not explicitly specify how participants with viewing durations within this interval should respond. Responses were analyzed according to the selected categories without inferring exact viewing durations. In the collapsed-category sensitivity analysis, the 2–4 and >4 h/day categories were combined and compared with the 0–1 h/day category.

### Three factor eating questionnaire

The Three-Factor Eating Questionnaire was first developed by Stunkard and Messick (23) as a 51-item measure of dietary restraint, disinhibition and hunger. Later revisions restructured the instrument around three different domains—cognitive restraint, uncontrolled eating and emotional eating—first in the 18-item form (24) and subsequently in the 21-item form used here, whose psychometric properties were established by Cappelleri et al. (25). The Turkish adaptation, validity, and reliability study of the 21-item version was conducted by Karakuş et al. (26). The TFEQ-R21 comprises three subscales: uncontrolled eating (9 items), cognitive restraint (6 items), and emotional eating (6 items). Item responses were coded and summed according to the instrument's scoring guidelines. Each subscale raw score was transformed to a 0–100 scale using the formula [(observed raw score – minimum possible raw score) / possible raw score range] × 100. Higher transformed scores indicate stronger expression of the corresponding eating behavior. In the present sample, Cronbach's alpha was 0.882 for UE, 0.821 for CR, and 0.927 for EE.

### Body appreciation scale-2

Body appreciation was assessed using the Body Appreciation Scale-2 (BAS-2), originally developed by Tylka and Wood-Barcalow (27) and adapted into Turkish by Anli et al. (28). The scale consists of 10 items rated on a five-point Likert scale ranging from 1 (“never”) to 5 (“always”). No items are reverse-coded. Item scores are summed to obtain a total score ranging from 10 to 50, with higher scores indicating greater body appreciation. In the present sample, Cronbach's alpha was 0.957.

### Statistical analysis

Analyses were conducted using SPSS version 24.0, except for the categorical sensitivity analysis of overall social media duration, which was performed in Python using statsmodels. Descriptive statistics were calculated for each variable, with continuous data reported as mean ± standard deviation and categorical data as frequencies and percentages. Data distributions were examined using the Kolmogorov–Smirnov test. Group differences were examined using Pearson's chi-square, Mann–Whitney U, or Kruskal–Wallis tests. Significant overall differences were further evaluated using Bonferroni-adjusted pairwise comparisons. Spearman's rank correlation coefficients was used to examine associations among age, BMI, uncontrolled eating, cognitive restraint, emotional eating, BAS-2, daily average social media use, and food-related video viewing durations.

Four-block hierarchical linear regression analyses were conducted separately for uncontrolled eating, emotional eating, and cognitive restraint. Covariates and their order of entry were selected a priori based on conceptual relevance: age, sex, and BMI were entered in Model 1; daily social media use and food-video viewing duration in Model 2; food-video genre in Model 3; and body appreciation in Model 4. Overall social media duration was entered in the primary regression models as an ordinal score coded 1–5 for < 1, 1–2, 2–4, 4–6, and >6 h/day, respectively. Its coefficient represents the outcome change per one-category increase, rather than per additional hour. Food-video viewing duration was entered as categorical indicator variables, with 0–1 h/day as the reference. In the categorical sensitivity analysis, the ordinal overall social media duration score was replaced by four indicator variables, using < 1 h/day as the reference. Model diagnostics included residual and Q–Q plots, studentised residuals, the studentised Breusch–Pagan test (Koenker version), Durbin–Watson statistics, Cook's distances, and variance inflation factors. HC3 heteroskedasticity-consistent standard errors and corresponding 95% confidence intervals were used for statistical inference. Results are presented as unstandardised coefficients (B) with 95% confidence intervals, standardized coefficients (β), *p*-values, R^2^, adjusted R^2^, and ΔR^2^. Individual regression coefficient *p*-values and 95% confidence intervals were based on HC3 heteroskedasticity-consistent standard errors in both the primary and sensitivity analyses. Overall model F tests and F-change tests associated with ΔR^2^ were conventional ordinary least-squares tests and were not adjusted for heteroskedasticity. The joint significance of the three food-video genre indicators in the final models was additionally assessed using an HC3-based robust Wald test. Conventional model and block-comparison tests were distinguished from robust coefficient and joint tests when interpreting the findings.

Sensitivity analyses were performed by additionally adjusting the final models for education, income, smoking, alcohol use, physical activity, and the presence of chronic disease. To assess the influence of the small >4 h/day group (*n* = 15), the final models were re-estimated after combining the 2–4 and >4 h/day categories (combined 2–4 and >4 h/day categories vs 0–1 h/day) and in leave-one-out analyses that sequentially excluded each participant in the >4 h/day group. An additional exploratory sensitivity analysis examined whether the associations of age, BMI, and body appreciation with cognitive restraint departed from linearity. This analysis assessed the sensitivity of the primary findings to the assumption of linear relationships between these continuous predictors and the outcome. Age, BMI, and BAS-2 scores were mean-centered, and their squared terms were entered simultaneously alongside their linear terms and all other predictors from the primary final model. Mean-centring facilitated interpretation of the linear terms at the sample means and reduced non-essential collinearity between the linear and quadratic terms. Statistical inference used HC3 heteroskedasticity-consistent standard errors and corresponding 95% confidence intervals. Multiple-testing adjustments were applied according to the analysis family. Following significant omnibus group comparisons, Bonferroni-adjusted pairwise comparisons were used to control the family-wise error rate within each set of *post hoc* comparisons. For the exploratory perceived-response analyses in Table 1, the Benjamini–Hochberg procedure was applied separately to five families of eight tests: associations with the matched eating-behavior subscale, food-video viewing duration, and general social media duration, and differences by sex and food-video genre. The resulting FDR-adjusted *p*-values are reported as *q*-values. No multiplicity adjustment was applied to the correlation matrix in Table 2 or to the primary and sensitivity regression analyses; these results were interpreted with consideration of the number of comparisons and the exploratory nature of the sensitivity analyses. All tests were two-sided, with statistical significance defined as *p* < 0.05 or, for FDR-adjusted analyses, *q* < 0.05.

**Table 1:** Perceived responses to food-related social media content and associations with eating behaviors and viewing patterns (*n* = 364).

Selected perceived response item	Agree, *n* (%)	Matched scale, ρ (q)	Food-video duration, ρ (q)	General social media duration, ρ (q)	Female vs male, median (IQR); q	Genre, H; ε^2^; q
Considering unhealthy behaviors after suggestive content	53 (14.6)	UE: 0.301 (< 0.001)	0.343 (< 0.001)	0.058 (0.313)	1.0 (1.0–3.0) vs 1.0 (1.0–3.0); 0.256	12.13; 0.025; 0.056
Guilt after content glorifying extreme thinness	81 (22.3)	EE: 0.422 (< 0.001)	0.274 (< 0.001)	0.046 (0.385)	2.0 (1.0–3.5) vs 1.0 (1.0–3.0); 0.002	5.89; 0.008; 0.154
Overeating after “cheat meal” content	71 (19.5)	EE: 0.397 (< 0.001)	0.248 (< 0.001)	0.090 (0.116)	2.0 (2.0–3.0) vs 2.0 (1.0–3.0); 0.303	9.81; 0.019; 0.056
Motivation to make healthier choices after healthy-eating content	233 (64.0)	CR: 0.209 (< 0.001)	0.057 (0.275)	0.140 (0.030)	4.0 (3.0–4.0) vs 4.0 (2.0–4.0); 0.010	9.73; 0.019; 0.056
Faster eating or larger portions after viewing	74 (20.3)	UE: 0.446 (< 0.001)	0.269 (< 0.001)	0.117 (0.050)	2.0 (2.0–3.0) vs 2.0 (1.0–3.0); 0.130	7.01; 0.011; 0.135
Difficulty distinguishing physical from psychological hunger	131 (36.0)	UE: 0.447 (< 0.001)	0.198 (< 0.001)	0.154 (0.026)	3.0 (2.0–4.0) vs 2.0 (1.0–4.0); 0.010	5.57; 0.007; 0.154
Food content triggering an uncontrolled eating urge or episode	115 (31.6)	UE: 0.501 (< 0.001)	0.249 (< 0.001)	0.129 (0.037)	3.0 (2.0–4.0) vs 2.0 (1.0–4.0); 0.136	4.03; 0.003; 0.258
Comparing one's eating style with people on social media	119 (32.7)	EE: 0.380 (< 0.001)	0.183 (< 0.001)	0.100 (0.089)	3.0 (2.0–4.0) vs 2.0 (1.0–4.0); 0.010	6.64; 0.010; 0.135

Agreement combines “agree” and “strongly agree.” Correlations are Spearman coefficients; sex and genre differences were tested using Mann–Whitney U and Kruskal–Wallis tests, respectively. Benjamini–Hochberg FDR correction was applied separately to each family of eight comparisons. Female values precede male values. UE, uncontrolled eating; EE, emotional eating; CR, cognitive restraint; IQR, interquartile range; ε^2^, rank epsilon-squared.

**Table 2:** Spearman correlations among age, BMI, eating behaviors, body appreciation, social media use, and food-related video viewing duration.

Variable	1	2	3	4	5	6	7	8
1. Uncontrolled eating	—							
2. Cognitive restraint	0.612^**^	—						
3. Emotional eating	0.716^**^	0.433^**^	—					
4. Body appreciation (BAS-2)	−0.303^**^	−0.165^**^	−0.272^**^	—				
5. BMI	0.142^**^	0.227^**^	0.200^**^	−0.155^**^	—			
6. Age	−0.080	0.227^**^	0.036	0.058	0.312^**^	—		
7. Daily average social media use category (ordinal score)	0.176^**^	−0.038	0.068	−0.161^**^	−0.140^**^	−0.347^**^	—	
8. Daily average food-related video viewing duration	0.285^**^	0.166^**^	0.259^**^	−0.175^**^	−0.052	−0.068	0.295^**^	—

^**^p < 0.01. BMI, body mass index; BAS-2, Body Appreciation Scale-2.

## Results

Table 3 shows the sociodemographic characteristics of participants. Of the participants, 75.5% were female, and the mean age was 29.3 ± 10.8 years. Most participants were single (74.7%), had a bachelor's degree (65.1%), and lived with family or relatives (68.1%). Current smoking and alcohol use were reported by 42.6% and 53.6% of participants, respectively, while 29.1% were moderately active. The mean BMI was 23.5 ± 3.8 kg/m^2^, and 63.7% were in the normal BMI category.

**Table 3:** Sociodemographic characteristics (*n* = 364).

Variable	*n* (%) or mean ±SD
Age (mean ± SD)	29.3 ± 10.8
Sex	
Female	275 (75.5)
Male	89 (24.5)
Educational status	
Primary/secondary	3 (0.8)
High school	38 (10.4)
Associate	52 (14.3)
Bachelor	237 (65.1)
Postgraduate	34 (9.3)
Marital status	
Single	272 (74.7)
Married	92 (25.3)
Employment	
Student	132 (36.3)
Employed	152 (41.8)
Unemployed	67 (18.4)
Retired	13 (3.6)
Monthly personal income (TRY)	
0–15,000	83 (22.8)
15,001–30,000	45 (12.4)
30,001–45,000	65 (17.9)
45,001–60,000	36 (9.9)
≥60,001	73 (20.1)
Prefer not to say	62 (17.0)
Living arrangement	
Alone	70 (19.2)
Friends	37 (10.2)
Family/relatives	248 (68.1)
Partner	6 (1.6)
Dormitory	3 (0.8)
**Presence of a chronic disease (Yes)**	128 (35.2)
**Current smoking (Yes)**	155 (42.6)
**Current alcohol use (Yes)**	195 (53.6)
Physical activity	
Sedentary	68 (18.7)
Light	114 (31.3)
Moderate	106 (29.1)
Active	46 (12.6)
Very active	30 (8.2)
Body weight, kg (mean ± SD)	67.7 ± 13.7
Height, cm (mean ± SD)	169.4 ± 9.0
BMI, kg/m^2^ (mean ± SD)	23.5 ± 3.8
BMI classification	
Underweight	21 (5.8)
Normal	232 (63.7)
Overweight	89 (24.5)
Obese	22 (6.0)

BMI, body mass index.

Table 4 presents the social media use and food-related content consumption of participants. Instagram was the most frequently used social media platform with 59.6%, and entertainment or pastime was the most commonly reported primary purpose of use with 60.7%. Daily social media use was most frequently 2–4 h (37.9%). Most participants watched food-related videos for 0–1 h/day (81.3%); fitness/diet content (37.9%) and restaurant/food reviews (31.6%) were the most frequently watched genres.

**Table 4:** Social media use and food-related content consumption (*n* = 364).

Variable	*n*	%
Most-used platform		
Instagram	217	59.6
TikTok	76	20.9
YouTube	40	11.0
X/Other	31	8.5
Daily social media use category (ordinal score)		
< 1 h	47	12.9
1–2 h	84	23.1
2–4 h	138	37.9
4–6 h	69	19.0
>6 h	26	7.1
Primary purpose of use		
Entertainment/pastime	221	60.7
Information/news	93	25.5
Social connection	34	9.3
Health/nutrition/fitness content	6	1.6
Multiple/other	6	1.6
Food-related content	4	1.1
Daily food-video viewing		
0–1 h	296	81.3
2–4 h	53	14.6
>4 h	15	4.1
Most-watched food-video genre		
Mukbang/tasting	43	11.8
Restaurant/food review	115	31.6
Recipes	68	18.7
Fitness/diet	138	37.9

Table 5 shows the participants' characteristics and study measures by most frequently watched food-video genre. There were a significant differences between genres for Sex (*p* < 0.001), age (*p* = 0.003), and membership in the combined 2–4 and >4 h/day food-video viewing categories (*p* = 0.002), whereas BMI did not show statistical differences (*p* = 0.323). Uncontrolled eating was highest in the mukbang/tasting group (*p* = 0.011), while cognitive restraint (*p* = 0.003) and emotional eating (*p* = 0.005) also differed significantly by genre.

**Table 5:** Participant characteristics and study measures by most frequently watched food-video genre (*n* = 364).

Characteristic	Mukbang/ tasting (*n* = 43)	Restaurant/ food review (*n* = 115)	Recipes (*n* = 68)	Fitness/ diet (*n* = 138)	*p*-value
Female, %	74.4	66.1	67.6	87.7	**< 0.001** ^**^
Age, years (mean ± SD)	26.5 ± 8.0	27.7 ± 8.5	28.6 ± 11.6	31.9 ± 12.3	**0.003** ^*^
BMI, kg/m^2^ (mean ± SD)	23.9 ± 4.3	23.0 ± 3.7	23.8 ± 4.0	23.7 ± 3.6	0.323
Combined 2–4 and >4 h/day food-video viewing categories, %	37.2	12.2	23.5	15.9	**0.002** ^*^
Uncontrolled eating — median (IQR)	59.3 (38.9–70.4)	44.4 (33.3–63.0)	51.9 (33.3–66.7)	40.7 (33.3–55.6)	**0.011** ^*^
Cognitive restraint — median (IQR)	44.4 (36.1–63.9)	44.4 (33.3–55.6)	55.6 (44.4–66.7)	50.0 (33.3–66.7)	**0.003** ^*^
Emotional eating — median (IQR)	50.0 (16.7–72.2)	38.9 (16.7–66.7)	55.6 (33.3–72.2)	38.9 (16.7–55.6)	**0.005** ^*^
Body appreciation (BAS-2) — median (IQR)	36.0 (28.5–41.0)	40.0 (30.0–41.0)	38.5 (30.0–40.2)	37.0 (31.0–40.0)	0.242

^*^p < 0.05, ^**^p < 0.001. Values are presented as median (interquartile range) or n (%). Continuous variables were compared using the Kruskal–Wallis test, and categorical variables were compared using Pearson's chi-square test. Significant pairwise differences were observed between the following groups: for sex, fitness/diet vs. restaurant/food review and recipes; for age, fitness/diet vs. mukbang/tasting and restaurant/food review; for the combined 2–4 and >4 h/day food-video viewing categories, mukbang/tasting vs. restaurant/food review and fitness/diet; for uncontrolled eating, mukbang/tasting vs. fitness/diet; for cognitive restraint, recipes vs. restaurant/food review; and for emotional eating, recipes vs. restaurant/food review and fitness/diet.

Table 1 presents the perceived responses to food-related social media content and associations with eating behaviors and viewing patterns. The most frequently endorsed response was motivation to make healthier choices after viewing healthy-eating content (64.0%), followed by difficulty distinguishing physical from psychological hunger (36.0%). Food content triggering an uncontrolled eating urge or episode showed the strongest association with uncontrolled eating ((ρ = 0.501, *q* < 0.001), while guilt after content glorifying extreme thinness showed the strongest association with emotional eating (ρ = 0.422, *q* < 0.001). Daily food-video viewing duration was positively associated with seven of the eight selected responses (ρ = 0.183–0.343, *q* < 0.001 for all), whereas associations with general social media duration were weaker and less consistent.

Table 2 shows the spearman correlations among age, BMI, eating behaviors, body appreciation, social media use, and food-related video viewing duration. Body appreciation was weakly negatively correlated with uncontrolled eating (r: −0.303, *p* < 0.01), emotional eating (r: −0.272, *p* < 0.01), and cognitive restraint (ρ: −0.165, *p* < 0.01). There was a positive weak correlation between daily food-video viewing duration and uncontrolled eating (r: 0.285, *p* < 0.01), emotional eating (r: 0.259, *p* < 0.01), and cognitive restraint (r: 0.166, *p* < 0.01), while there was a negative weak correlation between body appreciation (r: −0.175, *p* < 0.01).

Table 6 presents the hierarchical linear regression models for uncontrolled eating, emotional eating, and cognitive restraint; individual predictor estimates correspond to the final models (Model 4). Model 1, which included age, sex, and BMI, explained 3.4%, 10.3%, and 7.5% of the variance in uncontrolled eating, emotional eating, and cognitive restraint, respectively. Adding daily social media use and food-video viewing duration in Model 2 significantly increased the explained variance by 7.4% for uncontrolled eating (*p* < 0.001), 5.1% for emotional eating (*p* < 0.001), and 3.2% for cognitive restraint (*p* = 0.006). The addition of the most-watched food-video genre in Model 3 did not significantly improve the uncontrolled eating model (ΔR^2^ = 0.013, *p* = 0.156), but significantly increased the explained variance in emotional eating by 3.4% (*p* = 0.002) and cognitive restraint by 3.0% (*p* = 0.007). Adding body appreciation in Model 4 significantly increased the explained variance in uncontrolled eating by 4.0% (*p* < 0.001) and emotional eating by 3.2% (*p* < 0.001), but did not significantly improve the cognitive restraint model (ΔR^2^ = 0.009, *p* = 0.059). In the final models, viewing food videos for 2–4 h/day was independently associated with higher uncontrolled eating (β: 0.208, *p* < 0.001), emotional eating (β: 0.161, *p* = 0.001), and cognitive restraint (β: 0.103, *p* =0.045), whereas viewing for >4 h/day was associated with higher emotional eating (β: 0.118, *p* = 0.001) and cognitive restraint (β: 0.147, *p* = 0.006). Higher body appreciation was independently associated with lower uncontrolled eating (β: −0.212, *p* < 0.001) and emotional eating (β: −0.191, *p* < 0.001). Female sex (β: 0.224, *p* < 0.001) and higher BMI (β: 0.255, *p* < 0.001) were associated with higher emotional eating, whereas older age (β: 0.174, *p* < 0.001) and higher BMI (β: 0.138, *p* = 0.018) were associated with greater cognitive restraint. The final models explained 13.7%, 19.7%, and 12.2% of the adjusted variance in uncontrolled eating, emotional eating, and cognitive restraint, respectively. In the primary final models, the maximum variance inflation factor was 1.363. Durbin–Watson statistics were 2.111, 2.157, and 2.004 for uncontrolled eating, emotional eating, and cognitive restraint, respectively. Visual inspection of the Q–Q plots indicated approximate residual normality, with modest departures in the tails. Residual-vs.-fitted plots showed no pronounced systematic curvature for uncontrolled or emotional eating, although variation in residual spread was apparent for emotional eating, consistent with the significant Breusch–Pagan test. For cognitive restraint, the smoothed residual pattern showed curvature, suggesting that the primary linear specification did not fully capture the underlying relationships and supporting the exploratory nonlinear sensitivity analysis. Studentised Breusch–Pagan tests indicated heteroskedasticity in the emotional-eating model (*p* = 0.035), but not in the uncontrolled-eating (*p* = 0.525) or cognitive-restraint model (*p* = 0.657). HC3 heteroskedasticity-consistent inference was used for all outcomes. Maximum Cook's distances were 0.046, 0.030, and 0.050, respectively. One observation in the uncontrolled-eating model and none in the other models had an absolute externally studentised residual exceeding 3. Using 4/n as an influence-screening threshold, 22, 18, and 17 observations, respectively, were flagged; these flags were not treated as automatic exclusion criteria.

**Table 6:** Hierarchical linear regression models predicting eating behaviors.

Predictor	Uncontrolled eating B (95% CI)	β (*p*)	Emotional eating B (95% CI)	β (*p*)	Cognitive restraint B (95% CI)	β (*p*)
Age, years	−0.146 (−0.366, 0.073)	−0.070 (0.190)	−0.082 (−0.379, 0.216)	−0.030 (0.589)	0.355 (0.148, 0.562)	0.174 (< 0.001)
Female sex	1.628 (−4.160, 7.416)	0.031 (0.580)	15.221 (7.975, 22.467)	0.224 (< 0.001)	3.342 (−2.616, 9.301)	0.065 (0.270)
BMI, kg/m^2^	0.645 (−0.061, 1.351)	0.109 (0.072)	1.960 (1.046, 2.874)	0.255 (< 0.001)	0.799 (0.136, 1.461)	0.138 (0.018)
Daily social media use category (ordinal score)	0.716 (−1.456, 2.889)	0.035 (0.517)	−0.801 (−3.544, 1.941)	−0.030 (0.566)	−1.110 (−3.175, 0.955)	−0.055 (0.290)
Food-video viewing: 2–4 h/day	13.248 (6.472, 20.025)	0.208 (< 0.001)	13.318 (5.587, 21.049)	0.161 (0.001)	6.434 (0.124, 12.744)	0.103 (0.045)
Food-video viewing: >4 h/day	6.871 (−4.926, 18.669)	0.061 (0.252)	17.334 (6.666, 28.002)	0.118 (0.001)	16.376 (4.640, 28.113)	0.147 (0.006)
Genre: mukbang/tasting	6.508 (−0.741, 13.757)	0.093 (0.077)	4.318 (−4.997, 13.632)	0.048 (0.362)	−3.821 (−11.266, 3.624)	−0.056 (0.313)
Genre: restaurant/food review	3.054 (−2.348, 8.455)	0.063 (0.266)	7.383 (0.601, 14.165)	0.117 (0.032)	−3.885 (−9.345, 1.575)	−0.082 (0.162)
Genre: recipes	5.790 (−0.520, 12.099)	0.100 (0.071)	14.985 (7.433, 22.538)	0.200 (< 0.001)	6.473 (0.051, 12.895)	0.114 (0.047)
Body appreciation (BAS-2)	−0.561 (−0.867, −0.256)	−0.212 (< 0.001)	−0.657 (−1.023, −0.290)	−0.191 (< 0.001)	−0.259 (−0.542, 0.024)	−0.099 (0.072)
MODEL PERFORMANCE						
Model 1: R^2^; adjusted R^2^	0.034; 0.026		0.103; 0.095		0.075; 0.068	
Model 1: ΔR^2^ (p)	0.034 (0.006)		0.103 (< 0.001)		0.075 (< 0.001)	
Model 2: R^2^; adjusted R^2^	0.108; 0.093		0.153; 0.139		0.107; 0.092	
Model 2: ΔR^2^ (p)	0.074 (< 0.001)		0.051 (< 0.001)		0.032 (0.006)	
Model 3: R^2^; adjusted R^2^	0.121; 0.099		0.187; 0.167		0.137; 0.115	
Model 3: ΔR^2^ (p)	0.013 (0.156)		0.034 (0.002)		0.030 (0.007)	
Model 4: R^2^; adjusted R^2^	0.161; 0.137		0.219; 0.197		0.146; 0.122	
Model 4: ΔR^2^ (p)	0.040 (< 0.001)		0.032 (< 0.001)		0.009 (0.059)	
Final model F (p)	6.75 (< 0.001)		9.92 (< 0.001)		6.03 (< 0.001)	
Genre block, robust p	0.189		0.001		0.012	

Values for individual predictors are from the final model (Model 4); 95% confidence intervals are based on HC3 heteroskedasticity-robust standard errors. Model 1 included age, sex, and BMI. Model 2 additionally included daily average social media use and food-video viewing duration; Model 3 added most-watched food-video genre; Model 4 added body appreciation. Reference categories were male sex, food-video viewing 0–1 h/day, and fitness/diet genre. The maximum VIF was 1.36. B, unstandardised coefficient; SE, robust standard error; CI, confidence interval; β, standardized coefficient; ΔR^2^, change in explained variance. Individual coefficient *p*-values and 95% confidence intervals are HC3-robust. Overall model F tests and *p*-values associated with ΔR^2^ are conventional OLS tests. “Genre block, robust p” denotes the HC3-based joint Wald test of the three genre indicators in the final model. General social media use was coded as an ordinal score from 1 to 5; B represents the change per one-category increase.

Sensitivity analyses are presented in Supplementary Table S1. In the expanded covariate-adjusted models (Panel A), viewing food videos for 2–4 h/day remained associated with uncontrolled eating (B = 12.467, 95% CI 5.258–19.676; β = 0.196, *p* < 0.001) and emotional eating (B=11.507, 95% CI 3.255–19.758; β = 0.139, *p* = 0.006), but not cognitive restraint (B = 4.982, 95% CI −1.936–11.899; β = 0.080, *p* = 0.158). Viewing for >4 h/day remained associated with emotional eating (B = 16.751, 95% CI 5.869–27.633; β = 0.114, *p* = 0.003) and cognitive restraint (*B* = 14.647, 95% CI 2.908–26.386; β = 0.132, *p* = 0.015), but not uncontrolled eating (B = 7.213, 95% CI −4.237–18.664; β = 0.064, *p* = 0.216). Body appreciation was inversely associated with uncontrolled eating (B = −0.549, 95% CI −0.878–−0.219; β = −0.207, *p* = 0.001), emotional eating (B=-0.628, 95% CI −1.011–−0.246; β = −0.182, *p* = 0.001), and cognitive restraint (B=-0.303, 95% CI −0.603–−0.004; β = −0.117, *p* = 0.047).

When the 2–4 and >4 h/day categories were combined (Panel B), membership in the combined 2–4 and >4 h/day food-video viewing categories associated with uncontrolled eating (B = 11.958, 95% CI 5.681–18.236; β = 0.207, *p* < 0.001), emotional eating (B = 14.131, 95% CI 7.161–21.101; β = 0.189, *p* < 0.001), and cognitive restraint (B = 8.445, 95% CI 2.629–14.262; β = 0.149, *p* = 0.005). In leave-one-out analyses of the 15 participants reporting >4 h/day (Panel C), the coefficients for this category remained significant for emotional eating (B range 15.790–20.687; all p ≤ 0.004) and cognitive restraint (B range 14.122–19.575; all p ≤ 0.017), but remained non-significant for uncontrolled eating (B range 4.403–10.288; all p≥0.053). Estimates for this small subgroup should nevertheless be interpreted cautiously.

In the exploratory non-linear sensitivity model for cognitive restraint (Supplementary Table S1, Panel D), the quadratic terms for age (B = −0.017, 95% CI −0.029 to −0.005; *p* = 0.005), BMI (B=-0.188, 95% CI −0.294 to −0.082; *p* < 0.001), and body appreciation (B = −0.043, 95% CI −0.062 to −0.024; *p* < 0.001) were significant and negative, indicating concave relationships within the fitted model. The adjusted R^2^ was 0.209, compared with 0.122 in the primary linear model. Viewing food videos for >4 h/day remained associated with cognitive restraint (B = 18.535, 95% CI 7.582–29.488; p < 0.001), whereas the association for 2–4 h/day was no longer significant (B = 5.170, 95% CI −0.953–11.292; *p* = 0.098). Recipe viewing remained associated with cognitive restraint (B = 8.414, 95% CI 2.472–14.356; *p* = 0.006). Adjusted nonlinear relationships of age, BMI, and body appreciation with cognitive restraint, together with pointwise 95% confidence intervals, are presented in Supplementary Figure S1.

Modeling overall social media duration categorically did not materially alter the primary findings (Supplementary Table S1, Panel E). The duration indicators were not jointly significant for uncontrolled eating (F [4, 350] = 0.269, *p* = 0.898), emotional eating (F [4, 350]= 0.578, *p* = 0.678), or cognitive restraint (F [4, 350] = 0.885, *p* = 0.473). None of the individual category contrasts was statistically significant, and the associations that were statistically significant in the primary final models remained significant.

## Discussion

Eating has become a form of entertainment: social media users are no longer merely exposed to food images but repeatedly watch others prepare, evaluate, restrict, and consume food (29). Such content can simultaneously stimulate appetite through visual food cues, promote social comparison and dietary restraint, or motivate healthier choices, making its relationship with eating behavior more complex than a simple “screen-time effect” (2, 6, 17). Against this background, food-video viewing duration was associated with eating behaviors in the adjusted models, whereas no statistically significant associations were observed for overall social media duration. However, this pattern does not establish that food-specific exposure has a stronger association than total social media use, because the difference between these associations was not formally tested. Total daily social media use was not significantly associated with any of the three eating behaviors. Viewing food videos for 2–4 h/day was associated with higher uncontrolled and emotional eating in both the primary and expanded covariate-adjusted models. Its association with cognitive restraint, however, was model-dependent: it was statistically significant in the primary model but not after expanded covariate adjustment or in the non-linear sensitivity analysis. Viewing for >4 h/day was associated with emotional eating in the primary and expanded models, while its association with cognitive restraint remained statistically significant in the primary, expanded, and non-linear models. Genre contributed additional information for emotional eating and restraint—but not for uncontrolled eating—and, unexpectedly, recipe and restaurant-review content emerged as more relevant than mukbang after adjustment. In contrast, body appreciation was independently associated with lower uncontrolled and emotional eating and explained additional variance beyond exposure and genre. Viewed within a multidomain framework, these findings suggest that digital exposure, psychological characteristics, and nutrition-related behaviors may represent interconnected dimensions rather than isolated factors. However, the present cross-sectional data do not establish the direction of these relationships.

Although general social media use and food-specific exposure are often treated interchangeably, only food-video viewing contributed independently to the eating-behavior models, whereas total social media duration showed no association with any outcome. This pattern is consistent with Sanzari et al. (17), who found that the type of content consumed was more strongly related to body-image disturbance and binge-eating symptoms than either time spent online or the number of platforms used. Our findings suggest a content-specific pattern for uncontrolled and emotional eating among community-dwelling adults, whereas the association with cognitive restraint requires a more qualified interpretation, particularly for the 2–4 h/day viewing category. Food-related videos may represent a particularly salient exposure because they repeatedly combine appetitive visual cues with portion norms, dietary rules, appearance ideals, and emotionally charged messages about food (2, 5). Accordingly, null associations based solely on overall screen time should not be interpreted as evidence that social media is irrelevant to eating behavior; rather, broad duration measures may obscure the influence of the specific content encountered. Since the present study was cross-sectional, however, this association may also reflect selective exposure, whereby individuals with particular eating tendencies are more likely to seek out food-related videos and subsequently receive more of them through algorithmic recommendation.

The exposure pattern also differed across eating behaviors and model specifications. Viewing food videos for 2–4 h/day was associated with higher uncontrolled and emotional eating in both the primary and expanded covariate-adjusted models, whereas its association with cognitive restraint was significant only in the primary model. Viewing for >4 h/day was associated with emotional eating and cognitive restraint in both models, and the cognitive-restraint association also remained significant in the non-linear sensitivity analysis. For uncontrolled eating, the exploratory perceived-response findings were compatible with, but did not establish, a cue-reactivity interpretation: reporting that food content triggered an uncontrolled eating urge showed the strongest association with uncontrolled eating, followed by difficulty distinguishing physical from psychological hunger. This accords with evidence that visual food cues elicit craving and predict subsequent intake and that repeated cue–food pairings can transfer incentive value to the cues themselves (6, 7). By contrast, guilt after content glorifying extreme thinness, overeating after “cheat meal” content, and comparing one's eating with that of others were most closely related to emotional eating, a pattern compatible with an affect-regulation interpretation consistent with Macht's model (12), although these correlations do not demonstrate this pathway. A scoping review of mukbang research reported that viewers often describe watching others eat as a source of companionship or emotional comfort, although loneliness itself was rarely measured in the studies reviewed (11). Cognitive restraint was positively associated with both viewing categories in the primary model, but the association for 2–4 h/day was not statistically significant in the expanded or non-linear models. Its correlation with the reported motivation to make healthier food choices should therefore be considered separately from the model-dependent association with viewing duration. However, this finding should not necessarily be interpreted as evidence of successful self-regulation, as cognitive restraint was moderately correlated with both uncontrolled and emotional eating in the present sample. The distinction between flexible and rigid control is therefore particularly relevant: prescriptive content that moralizes certain foods or promotes strict dietary rules may foster rigid rather than adaptive restraint, which can coexist with disinhibited eating (13). Finally, the lack of a significant association with uncontrolled eating among the heaviest viewers should not be interpreted as evidence of a protective or satiating effect. Although repeated exposure to food images may induce sensory-specific satiety (30, 31), the small size of this exposure subgroup limits the precision of the estimate and precludes firm conclusions about whether the finding reflects such a mechanism or insufficient statistical power.

Content genre provided a second, more nuanced layer of information. Although uncontrolled eating was highest among mukbang/tasting viewers in the unadjusted comparison, genre did not significantly improve the uncontrolled-eating model after age, sex, BMI, and viewing duration were considered. Mukbang viewers were also the group most likely to report food-video viewing in the 2–4 or >4 h/day categories, suggesting that the initial difference may have reflected greater exposure rather than a unique association with mukbang. This is compatible with evidence linking problematic or addiction-like mukbang engagement—not merely exposure—to disordered eating (10, 14), as well as evidence of no association between mukbang viewing frequency and emotional, external, or restrained eating in a small adult sample (32). More unexpectedly, recipe viewers reported higher emotional eating and cognitive restraint, and restaurant-review viewers reported higher emotional eating, relative to fitness/diet viewers. Existing research on culinary videos has focused mainly on food choice, cooking practices, and skill acquisition rather than eating-behavior traits (1). Similarly, an experiment conducted in adolescents found that short culinary videos altered liking of the foods portrayed, subsequent food choice, and intentions to prepare or eat them (33), indicating that such content can shape what viewers select rather than acting solely through immediate hunger. Recipe and restaurant-review videos may therefore operate not only through immediate appetite but also by encouraging viewers to prepare, purchase, or keep highly palatable foods within reach.

Beyond exposure duration and genre, body appreciation emerged as an independent correlate of eating behavior. In the hierarchical regression analyses, adding body appreciation explained more additional variance than adding content genre for uncontrolled eating, but slightly less for emotional eating. Body appreciation remained inversely associated with both outcomes in the final models. In the primary linear model, body appreciation was not significantly associated with cognitive restraint; however, the exploratory nonlinear sensitivity analysis suggested that this relationship may not be adequately represented by a single linear coefficient. Body appreciation involves accepting and respecting the body while filtering harmful appearance-related messages (18), and within the acceptance model it supports eating guided by internal hunger and satiety signals rather than external demands (19). It was observed that unconditional permission to eat may mediate the relationship between body appreciation and reduced eating pathology (34), while body appreciation has prospectively predicted the continued absence of core eating-disorder symptoms (20) and has been identified as a consistent protective factor in the wider social media literature (35). Therefore, a construct operating through acceptance and internal regulation may be more closely connected with lower loss-of-control and affect-driven eating than with deliberate restraint, as observed here. Importantly, body appreciation did not differ across genres and was only weakly correlated with food-video duration, indicating that it was not simply a proxy for lighter exposure. Given the cross-sectional design, however, these findings are consistent with—rather than proof of—a protective role.

The non-linear sensitivity analysis suggested departures from linearity in the associations of age, BMI, and body appreciation with cognitive restraint. The negative quadratic terms indicated concave fitted relationships, meaning that the direction and magnitude of these associations may vary across predictor values. Although the non-linear model had a higher adjusted R^2^, this exploratory finding requires confirmation in independent samples. The primary linear model was retained to report the a priori analytical specification consistently across the three outcomes, whereas the quadratic model was treated as an exploratory sensitivity analysis. However, retaining this model does not establish the adequacy of a linear specification for cognitive restraint. The non-linear findings suggest that the associations of age, BMI, and body appreciation with this outcome may not be fully captured by single linear coefficients; consequently, the primary estimates should be interpreted alongside the non-linear results and regarded as model-dependent. Associations involving cognitive restraint varied across model specifications. Viewing food videos for 2–4 h/day was associated with cognitive restraint in the primary model, but this association was not statistically significant after additional covariate adjustment or in the non-linear sensitivity model. Body appreciation was not significantly associated with cognitive restraint in the primary model, whereas an inverse association emerged after additional adjustment and a non-linear relationship was observed in the quadratic model. These findings indicate that associations with cognitive restraint should be interpreted as model-dependent rather than consistently supported across analyses.

The findings also argue against portraying food-related social media content as uniformly harmful. Almost two-thirds of participants reported feeling motivated to make healthier choices after viewing healthy-eating content, whereas responses describing guilt, overeating, or loss of control were endorsed by smaller proportions. Nevertheless, these less common adverse responses showed the strongest relationships with eating-behavior scores, suggesting that risk may be concentrated among susceptible viewers rather than distributed equally across all users. Demographic associations followed patterns reported in previous studies: women showed higher emotional eating, higher BMI was related to emotional eating and cognitive restraint, and older age was related to greater restraint (25, 36).

From a practical perspective, these findings may help inform integrated strategies that move beyond general recommendations to reduce screen time and instead address the nature of food-related content that individuals encounter and how they respond to it. Such strategies could combine digital health literacy and responsible engagement with food-related content, including greater awareness of persuasive, appearance-focused, or highly restrictive messages, with approaches that support positive body image and body appreciation. Where appropriate, emotional-regulation support may also help individuals recognize emotional triggers related to eating and distinguish them from physiological hunger, while evidence-based nutrition education may provide a counterbalance to misleading or overly prescriptive dietary messages encountered online. Importantly, these elements may be more appropriately considered as complementary components of an integrated approach rather than being addressed in isolation. The potential value of such integrated approaches should be evaluated in longitudinal and intervention studies before firm clinical or public-health recommendations are made.

The study has some limitations. First, the cross-sectional design precludes causal or temporal interpretations. People who eat in response to cues or emotion may seek out food content, and recommendation systems then increase supply once that preference is registered (5), producing exactly the association we report without any causal contribution from viewing. Our data cannot separate selection from exposure, and the models should be read as one consistent ordering rather than a demonstrated sequence. Second, exposure was self-reported using broad categories, with relatively few participants in the higher-exposure groups, limiting the precision of these estimates. In addition, recording only the most-watched genre may not fully reflect participants' mixed viewing habits. Third, height and weight were self-reported, which may have introduced measurement bias and weakened the observed associations between BMI and eating behaviors. Fourth, convenience and snowball sampling in a single city resulted in a predominantly young, female, university-educated sample, limiting the generalisability of the findings to broader populations. In addition, emotional eating was assessed as a specific eating-behavior construct, whereas broader emotional wellbeing, mood, or psychological health was not directly measured. Therefore, the observed associations involving emotional eating should not be interpreted as reflecting participants' overall emotional wellbeing. Dietary intake, diet quality, and quality of life were also not directly assessed; therefore, the observed eating-behavior associations cannot be assumed to reflect differences in actual food consumption, nutritional quality, or overall quality of life. Finally, the models explained only a modest proportion of the variance, suggesting that unmeasured psychological, behavioral, and environmental factors may also influence eating behaviors. Furthermore, the broad and differently defined exposure categories limited exact comparisons between total social media use and food-video viewing, and residual viewing-time inconsistencies cannot be excluded. Conceptual overlap between response items and TFEQ-R21 subscales, and their assessment in the same self-report questionnaire, may have inflated the correlations between them.

However, the study has several strengths. General social media use and exposure to food-related content were assessed separately, enabling their distinct associations with eating behaviors to be examined. The study included community-dwelling adults from a broad age range and used validated Turkish instruments. The hierarchical modeling approach evaluated the incremental contribution of different conceptual domains, with robust statistical methods and adjustment for relevant confounders. Moreover, examining body appreciation as a predictor of eating behavior offers a relatively novel perspective within the social media literature.

## Conclusion

Among adults living in Istanbul, Türkiye, food-video viewing duration was associated with uncontrolled and emotional eating, whereas overall social media duration was not significantly associated with the assessed eating behaviors. Findings for cognitive restraint were model-dependent: the association with viewing for 2–4 h/day was statistically significant in the primary model but not after expanded covariate adjustment or in the nonlinear sensitivity analysis, whereas the association with viewing for >4 h/day remained significant across these models. Estimates for the >4 h/day category nevertheless require caution because of the small subgroup size. Genre contributed additional explained variance for emotional eating and cognitive restraint in the primary models. Body appreciation was inversely associated with uncontrolled and emotional eating and contributed additional explained variance beyond the preceding predictor blocks. Longitudinal studies with continuous exposure measurement and dietary intake data are needed to establish temporal direction and clarify these associations.

## Data Availability

The raw data supporting the conclusions of this article will be made available by the authors, without undue reservation.
