# Supplementary material for: Watching what others eat: exposure to social-media eating content, body appreciation, and eating behaviors in Turkish adults
Source: Front Public Health. 2026 Oct 22;14:1963635. doi: 10.3389/fpubh.2026.1963635 (PMC13736711; doi:10.3389/fpubh.2026.1963635)
Supplement: Supplementary file 1 [file Supplementary_File_1.pdf]

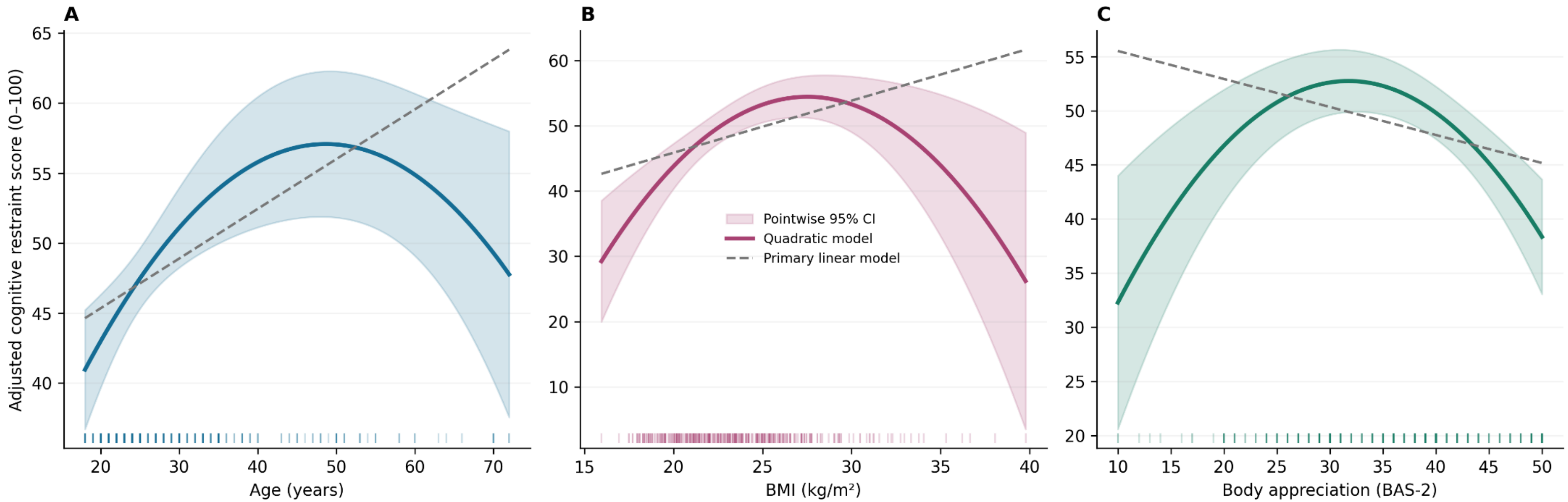

Supplementary Figure S1. Adjusted fitted relationships of age (A), BMI (B), and body appreciation (BAS-2; C) with cognitive restraint. Solid curves represent the quadratic sensitivity model containing mean-centred age, BMI, and BAS-2 scores and their squared terms, together with sex, daily social media use, food-video viewing category, and food-video genre.

Supplementary Table S1. Sensitivity Analyses for Hierarchical Regression Models

Panel A Expanded covariate adjusted models

Predictor	Uncontrolled eating B (95% CI)	beta (p)	Emotional eating B (95% CI)	beta (p)	Cognitive restraint B (95% CI)	beta (p)
**Primary model variables**						
Age, years	-0.327 (-0.599, -0.055)	-0.157 (0.019)	-0.227 (-0.586, 0.133)	-0.084 (0.216)	0.248 (-0.006, 0.502)	0.121 (0.055)
Female sex	1.959 (-4.606, 8.524)	0.037 (0.558)	15.719 (7.500, 23.939)	0.231 (<0.001)	3.633 (-2.881, 10.146)	0.071 (0.273)
BMI, kg/m2	0.621 (-0.140, 1.382)	0.105 (0.109)	2.074 (1.136, 3.012)	0.270 (<0.001)	0.818 (0.121, 1.516)	0.141 (0.022)
Daily social media use	0.018 (-2.554, 2.590)	0.001 (0.989)	-0.775 (-3.910, 2.360)	-0.029 (0.627)	-0.872 (-3.190, 1.446)	-0.043 (0.460)
Food-video viewing 2-4 h/day	12.467 (5.258, 19.676)	0.196 (<0.001)	11.507 (3.255, 19.758)	0.139 (0.006)	4.982 (-1.936, 11.899)	0.080 (0.158)
Food-video viewing >4 h/day	7.213 (-4.237, 18.664)	0.064 (0.216)	16.751 (5.869, 27.633)	0.114 (0.003)	14.647 (2.908, 26.386)	0.132 (0.015)
Genre: mukbang/tasting	6.540 (-1.088, 14.169)	0.094 (0.093)	3.177 (-6.649, 13.002)	0.035 (0.525)	-3.399 (-11.212, 4.414)	-0.050 (0.393)
Genre: restaurant/food review	3.967 (-1.849, 9.784)	0.082 (0.181)	7.433 (0.337, 14.528)	0.118 (0.040)	-3.398 (-9.222, 2.426)	-0.072 (0.252)
Genre: recipes	7.523 (0.780, 14.266)	0.131 (0.029)	15.764 (7.497, 24.032)	0.210 (<0.001)	6.815 (0.080, 13.550)	0.120 (0.047)
Body appreciation (BAS-2)	-0.549 (-0.878, -0.219)	-0.207 (0.001)	-0.628 (-1.011, -0.246)	-0.182 (0.001)	-0.303 (-0.603, -0.004)	-0.117 (0.047)
**Additional covariates**						
Education: associate	-4.920 (-15.938, 6.099)	-0.077 (0.380)	-10.808 (-25.499, 3.882)	-0.129 (0.149)	-6.490 (-17.382, 4.403)	-0.103 (0.242)

Predictor	Uncontrolled eating B (95% CI)	beta (p)	Emotional eating B (95% CI)	beta (p)	Cognitive restraint B (95% CI)	beta (p)
Education: bachelor	-10.126 (-19.624, -0.628)	-0.215 (0.037)	-9.405 (-21.800, 2.990)	-0.153 (0.136)	-5.331 (-14.948, 4.287)	-0.115 (0.276)
Education: postgraduate	-6.273 (-18.247, 5.701)	-0.081 (0.304)	2.968 (-11.771, 17.707)	0.030 (0.692)	-6.116 (-18.532, 6.299)	-0.081 (0.333)
Income: 15,001-30,000 TRY	-0.356 (-8.081, 7.370)	-0.005 (0.928)	-4.566 (-14.336, 5.204)	-0.051 (0.359)	-1.284 (-9.600, 7.033)	-0.019 (0.762)
Income: 30,001-45,000 TRY	2.331 (-5.036, 9.697)	0.040 (0.534)	3.241 (-5.911, 12.392)	0.042 (0.487)	6.158 (-1.125, 13.441)	0.107 (0.097)
Income: 45,001-60,000 TRY	4.033 (-6.608, 14.675)	0.054 (0.456)	6.075 (-6.957, 19.106)	0.062 (0.360)	9.843 (0.492, 19.195)	0.133 (0.039)
Income: >=60,001 TRY	0.096 (-7.492, 7.684)	0.002 (0.980)	-2.140 (-11.919, 7.638)	-0.029 (0.667)	2.995 (-4.615, 10.604)	0.054 (0.439)
Income: undisclosed	-6.561 (-15.116, 1.995)	-0.110 (0.132)	-6.491 (-16.053, 3.070)	-0.084 (0.183)	2.857 (-4.822, 10.535)	0.049 (0.465)
Current smoking	-2.033 (-7.270, 3.205)	-0.045 (0.446)	-5.815 (-12.232, 0.602)	-0.098 (0.076)	0.440 (-4.566, 5.447)	0.010 (0.863)
Current alcohol use	1.030 (-4.215, 6.275)	0.023 (0.700)	7.549 (1.327, 13.771)	0.129 (0.018)	-4.013 (-8.902, 0.877)	-0.091 (0.107)
Physical activity: light	3.312 (-3.654, 10.279)	0.068 (0.350)	4.721 (-3.697, 13.140)	0.075 (0.271)	2.912 (-3.056, 8.881)	0.061 (0.338)
Physical activity: moderate	1.848 (-5.394, 9.089)	0.037 (0.616)	4.189 (-4.616, 12.993)	0.065 (0.350)	4.689 (-2.055, 11.432)	0.096 (0.172)
Physical activity: active	-0.980 (-10.318, 8.357)	-0.014 (0.836)	2.239 (-9.584, 14.063)	0.025 (0.710)	5.150 (-3.934, 14.233)	0.078 (0.266)
Physical activity: very active	1.093 (-8.948, 11.133)	0.013 (0.831)	4.385 (-6.569, 15.339)	0.041 (0.432)	5.221 (-4.046, 14.489)	0.065 (0.269)

Predictor	Uncontrolled eating B (95% CI)	beta (p)	Emotional eating B (95% CI)	beta (p)	Cognitive restraint B (95% CI)	beta (p)
Chronic disease	0.754 (-4.383, 5.892)	0.016 (0.773)	0.637 (-5.751, 7.025)	0.010 (0.845)	-2.816 (-7.790, 2.157)	-0.061 (0.266)
**Model performance**						
**R2; adjusted R2**	**0.199; 0.140**	**-**	**0.269; 0.214**	**-**	**0.184; 0.123**	**-**
**F (p)**	**3.36 (<0.001)**	**-**	**4.96 (<0.001)**	**-**	**3.04 (<0.001)**	**-**

All estimates are from models including the complete set of predictors shown. HC3 heteroskedasticity-consistent 95% confidence intervals and p-values are reported. Reference categories were male sex, food-video viewing 0-1 h/day, fitness/diet genre, education up to high school, monthly income 0-15,000 TRY, no current smoking, no current alcohol use, sedentary physical activity, and no chronic disease. The maximum VIF was 3.01.

Panel B Collapsed food video viewing category models

Predictor	Uncontrolled eating B (95% CI)	beta (p)	Emotional eating B (95% CI)	beta (p)	Cognitive restraint B (95% CI)	beta (p)
Age, years	-0.146 (-0.366, 0.074)	-0.070 (0.191)	-0.082 (-0.379, 0.216)	-0.030 (0.590)	0.355 (0.149, 0.562)	0.174 (<0.001)
Female sex	1.866 (-3.908, 7.639)	0.036 (0.526)	15.072 (7.903, 22.241)	0.222 (<0.001)	2.973 (-2.926, 8.872)	0.058 (0.322)
BMI, kg/m2	0.675 (-0.024, 1.374)	0.114 (0.058)	1.942 (1.034, 2.850)	0.253 (<0.001)	0.753 (0.096, 1.410)	0.130 (0.025)
Daily social media use	0.675 (-1.503, 2.853)	0.033 (0.543)	-0.775 (-3.513, 1.963)	-0.029 (0.578)	-1.045 (-3.107, 1.017)	-0.052 (0.320)
Food-video viewing: combined 2–4 and >4 h/day categories	11.958 (5.681, 18.236)	0.207 (<0.001)	14.131 (7.161, 21.101)	0.189 (<0.001)	8.445 (2.629, 14.262)	0.149 (0.005)
Genre: mukbang/tasting	6.098 (-1.066, 13.261)	0.088 (0.095)	4.576 (-4.681, 13.833)	0.051 (0.332)	-3.182 (-10.607, 4.244)	-0.047 (0.400)
Genre: restaurant/food review	2.928 (-2.449, 8.306)	0.061 (0.285)	7.462 (0.721, 14.202)	0.119 (0.030)	-3.690 (-9.143, 1.763)	-0.078 (0.184)

Predictor	Uncontrolled eating B (95% CI)	beta (p)	Emotional eating B (95% CI)	beta (p)	Cognitive restraint B (95% CI)	beta (p)
Genre: recipes	5.999 (-0.325, 12.323)	0.104 (0.063)	14.854 (7.323, 22.385)	0.198 (<0.001)	6.147 (-0.222, 12.516)	0.109 (0.059)
Body appreciation (BAS-2)	-0.545 (-0.844, -0.245)	-0.205 (<0.001)	-0.667 (-1.026, -0.309)	-0.194 (<0.001)	-0.284 (-0.558, -0.010)	-0.109 (0.042)
**Model performance**						
**R2; adjusted R2**	**0.158; 0.137**	**-**	**0.219; 0.199**	**-**	**0.140; 0.118**	**-**
**F (p)**	**7.39 (<0.001)**	**-**	**11.02 (<0.001)**	**-**	**6.38 (<0.001)**	**-**

The original 2–4 and >4 h/day food-video viewing categories were combined and compared with the original 0–1 h/day category. No exact viewing durations were inferred, and participants' responses were not reassigned to address the undefined interval between 1 and 2 h/day.

Panel C Leave one out analysis of the >4 h/day group

**Sequential exclusion**	**Uncontrolled eating B (95% CI); p**	**Emotional eating B (95% CI); p**	**Cognitive restraint B (95% CI); p**
>4 h participant 1	4.403 (-6.964, 15.770); 0.447	16.817 (5.530, 28.104); 0.004	15.295 (2.994, 27.597); 0.015
>4 h participant 2	10.288 (-0.141, 20.718); 0.053	20.687 (11.785, 29.589); <0.001	19.575 (8.965, 30.185); <0.001
>4 h participant 3	7.236 (-5.232, 19.704); 0.254	16.508 (5.490, 27.525); 0.003	16.951 (4.652, 29.251); 0.007
>4 h participant 4	6.842 (-5.780, 19.463); 0.287	18.199 (6.950, 29.449); 0.002	15.855 (3.363, 28.347); 0.013
>4 h participant 5	5.320 (-6.836, 17.477); 0.390	17.163 (5.781, 28.545); 0.003	14.122 (2.572, 25.671); 0.017
>4 h participant 6	8.242 (-4.074, 20.558); 0.189	17.739 (6.439, 29.039); 0.002	15.727 (3.285, 28.170); 0.013
>4 h participant 7	5.973 (-6.483, 18.430); 0.346	17.517 (6.169, 28.865); 0.003	15.707 (3.181, 28.232); 0.014
>4 h participant 8	6.277 (-6.292, 18.847); 0.327	15.790 (5.072, 26.507); 0.004	15.074 (2.936, 27.211); 0.015
>4 h participant 9	7.284 (-4.987, 19.556); 0.244	17.866 (6.973, 28.758); 0.001	16.639 (4.413, 28.864); 0.008
>4 h participant 10	7.821 (-4.565, 20.208); 0.215	17.842 (6.654, 29.030); 0.002	15.355 (3.092, 27.618); 0.014
>4 h participant 11	8.085 (-4.644, 20.815); 0.212	17.517 (5.794, 29.240); 0.004	17.972 (5.550, 30.394); 0.005
>4 h participant 12	6.502 (-5.986, 18.991); 0.307	18.172 (7.055, 29.289); 0.001	17.924 (5.880, 29.969); 0.004
>4 h participant 13	4.984 (-6.647, 16.615); 0.400	15.867 (5.345, 26.388); 0.003	14.958 (3.091, 26.825); 0.014
>4 h participant 14	6.580 (-5.742, 18.903); 0.294	16.164 (5.509, 26.819); 0.003	16.799 (4.573, 29.025); 0.007
>4 h participant 15	7.895 (-4.438, 20.228); 0.209	17.059 (5.951, 28.166); 0.003	18.058 (6.122, 29.994); 0.003
**Range across 15 analyses**	**B 4.403 to 10.288; p 0.053 to 0.447**	**B 15.790 to 20.687; p <0.001 to 0.004**	**B 14.122 to 19.575; p <0.001 to 0.017**

Each analysis excluded one of the 15 participants in the >4 h/day group and re-estimated the primary final model; therefore, the focal category contained 14 participants in each iteration. Participant identifiers are not shown. HC3 robust 95% confidence intervals and p-values are reported.

Panel D Nonlinear sensitivity model for cognitive restraint

Predictor	B (95% CI)	p-value
Age, years	0.663 (0.308, 1.018)	<0.001
Female sex	4.061 (-1.574, 9.697)	0.157
BMI, kg/m2	1.518 (0.785, 2.252)	<0.001
Daily social media use	-0.238 (-2.369, 1.894)	0.827
Food-video viewing 2-4 h/day	5.170 (-0.953, 11.292)	0.098
Food-video viewing >4 h/day	18.535 (7.582, 29.488)	<0.001
Genre: mukbang/tasting	-1.921 (-9.316, 5.473)	0.610
Genre: restaurant/food review	-3.262 (-8.477, 1.952)	0.219
Genre: recipes	8.414 (2.472, 14.356)	0.006
Body appreciation (BAS-2)	-0.411 (-0.691, -0.131)	0.004
Age squared	-0.017 (-0.029, -0.005)	0.005
BMI squared	-0.188 (-0.294, -0.082)	<0.001
Body appreciation squared	-0.043 (-0.062, -0.024)	<0.001
**Model performance**		
**R2; adjusted R2**	**0.237; 0.209**	**-**
**F (p)**	**8.37 (<0.001)**	**-**

Age, BMI, and BAS-2 were mean-centred before their quadratic terms were calculated. All other predictors and reference categories were identical to the primary final model. HC3 robust 95% confidence intervals and p-values are reported. B, unstandardised regression coefficient; BAS-2, Body Appreciation Scale-2; beta, standardised regression coefficient; BMI, body mass index; CI, confidence interval; HC3, heteroskedasticity-consistent covariance estimator; R2, coefficient of determination; VIF, variance inflation factor.

Panel E Categorical sensitivity analysis of overall social media duration

**Predictor**	**Uncontrolled eating B (95% CI); p**	**Emotional eating B (95% CI); p**	**Cognitive restraint B (95% CI); p**
Age, years	-0.145 (-0.365, 0.075); 0.195	-0.091 (-0.385, 0.202); 0.541	0.349 (0.147, 0.551); <0.001
Female sex	1.525 (-4.363, 7.414); 0.611	15.549 (8.215, 22.883); <0.001	3.746 (-2.315, 9.808); 0.225
BMI, kg/m²	0.656 (-0.058, 1.371); 0.072	1.964 (1.038, 2.890); <0.001	0.794 (0.115, 1.472); 0.022
Social media use: 1–2 h/day	-0.332 (-7.303, 6.639); 0.925	4.529 (-4.348, 13.405); 0.316	4.589 (-2.934, 12.112); 0.231
Social media use: 2–4 h/day	1.139 (-5.186, 7.464); 0.723	-0.540 (-8.847, 7.766); 0.898	0.239 (-6.744, 7.222); 0.946
Social media use: 4–6 h/day	3.332 (-4.572, 11.237); 0.408	1.287 (-9.227, 11.802); 0.810	-0.259 (-8.056, 7.537); 0.948
Social media use: >6 h/day	0.135 (-10.451, 10.721); 0.980	-2.422 (-15.358, 10.514); 0.713	-3.062 (-14.107, 7.984); 0.586
Food-video viewing: 2–4 h/day	13.247 (6.413, 20.080); <0.001	13.370 (5.578, 21.162); <0.001	6.502 (0.120, 12.884); 0.046
Food-video viewing: >4 h/day	6.883 (-5.133, 18.898); 0.261	16.754 (5.873, 27.635); 0.003	16.486 (4.632, 28.339); 0.007
Genre: mukbang/tasting	6.470 (-0.844, 13.784); 0.083	4.277 (-5.149, 13.703); 0.373	-3.890 (-11.273, 3.493); 0.301
Genre: restaurant/food review	2.887 (-2.560, 8.333); 0.298	7.467 (0.637, 14.297); 0.032	-3.605 (-9.181, 1.971); 0.204
Genre: recipes	5.917 (-0.530, 12.364); 0.072	15.266 (7.605, 22.927); <0.001	6.847 (0.252, 13.442); 0.042
Body appreciation (BAS-2)	-0.554 (-0.862, -0.246); <0.001	-0.667 (-1.033, -0.301); <0.001	-0.273 (-0.555, 0.009); 0.058
R^2; adjusted R^2	0.162; 0.131	0.224; 0.195	0.152; 0.121
Social media block robust F (4, 350)	0.269	0.578	0.885
Social media block robust p	0.898	0.678	0.473

Reference categories: overall social media use <1 h/day; male sex; food-video viewing 0–1 h/day; fitness/diet genre. Overall social media group sizes were 47, 84, 138, 69, and 26 for <1, 1–2, 2–4, 4–6, and >6 h/day, respectively. Outcomes were transformed to the 0–100 scale. B, unstandardised coeff
